# Electrostatics as a Guiding Principle in Understanding and Designing Enzymes

**DOI:** 10.1021/acs.jctc.3c01395

**Published:** 2024-02-27

**Authors:** J. Javier Ruiz-Pernía, Katarzyna Świderek, Joan Bertran, Vicent Moliner, Iñaki Tuñón

**Affiliations:** † Departament de Química Física, Universitat de València, 46100 Burjassot, Spain; ‡ Biocomp group, Institute of Advanced Materials (INAM), Universitat Jaume I, 12071 Castellón Spain; § Departament de Química, Universitat Autònoma de Barcelona, 08193 Bellaterra, Spain

## Abstract

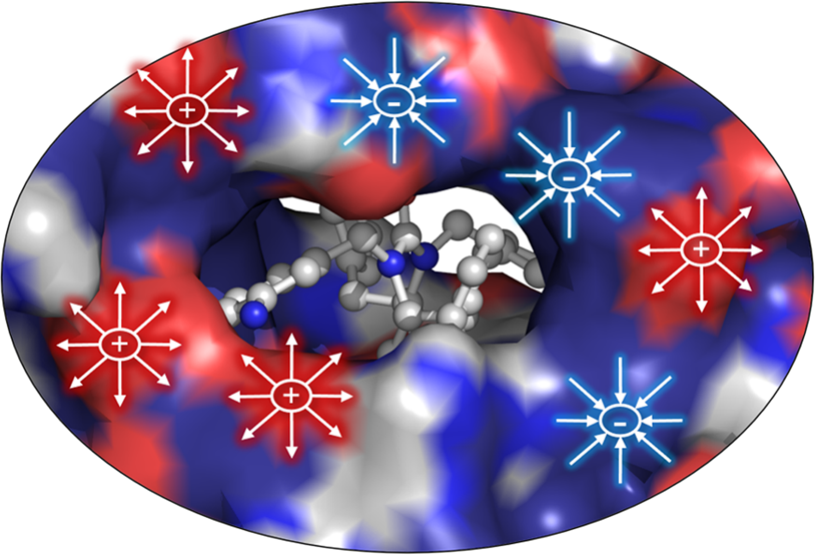

Enzyme design faces challenges related to the implementation of the basic principles that govern the catalytic activity in natural enzymes. In this work, we revisit basic electrostatic concepts that have been shown to explain the origin of enzymatic efficiency like preorganization and reorganization. Using magnitudes such as the electrostatic potential and the electric field generated by the protein, we explain how these concepts work in different enzymes and how they can be used to rationalize the consequences of point mutations. We also discuss examples of protein design in which electrostatic effects have been implemented. For the near future, molecular simulations, coupled with the use of machine learning methods, can be used to implement electrostatics as a guiding principle for enzyme designs.

## 1 Introduction

Enzymes are biological catalysts, mostly proteins, that can speed up chemical reactions by several orders of magnitude to fulfill the kinetic requirements for life. These biocatalysts can work under mild conditions of pressure and temperature and often present a high degree of selectivity, which makes them an attractive choice also as catalysts for industrial applications. However, the use of enzymes in industrial processes is still far from being general, mainly because of two limitations. On one side, enzymes need to be stable enough in those pH, temperature, and composition conditions that are used in the industrial process. But more importantly, the catalogue of chemical reactions catalyzed by enzymes at a reasonable level of efficiency required in industry is limited, in such a way that most industrial processes still do not have a natural enzyme that could catalyze them.

The limitations for the use of enzymes in industry can be overcome through protein engineering techniques that could confer the desired stability and catalytic properties to an initial protein scaffold. The engineering processes can be classified according to the strategies used to tailor the target properties into the protein scaffold, which can be based on the use of directed evolution^1^ or on a rational designing process.^2^ Directed evolution mimics natural evolution in a process that selects mutations that produce successful protein variants. New proteins with new desired functions can be obtained after several cycles of mutations or recombining protein fragments. This strategy does not require structural knowledge of the biocatalysts or a rationalization of its catalytic efficiency, because the mutations are performed randomly. In many cases, these mutations appear in positions relatively far from the active site^3^ which could have a limited effect on the catalytic properties although still beneficial for other aspects, such as the thermal stability of the enzyme. Another limitation of directed evolution is that minimal catalytic activity is required to start the cycles of mutations and screening. Instead, the rational design process refers to the introduction of a few selected mutations on specific positions of the initial protein structure. The selection is usually based on an explicit rationalization of the effect of the selected residue on the catalytic properties of the protein. Nowadays, machine learning techniques have been also incorporated in the process of protein design.^4−6^ In this case, an algorithm obtained after training on a large data set can predict the properties of a protein based on a set of descriptors. For example, the three-dimensional structure of the protein can be nowadays predicted from its sequence, using algorithms trained on already existing structures.^7,8^ For a catalytic process, the selection of a particular protein scaffold to integrate an adequate active site can be performed using a deep-learning based approach,^9^ while mutations introduced to improve catalytic efficiency can be also selected integrating machine learning methods.^10^ The machine learning designing process does not require an explicit knowledge of how a particular residue affects the properties of the biocatalyst but a prediction of the consequences of its mutation. The rational contribution to this designing process is in the selection of the descriptors used by the algorithm in its prediction.

Either for a rational prediction of the consequences of a mutation on catalysis or for the selection of the predictors of the catalytic properties in a machine learning designing process, a certain degree of guiding is needed. Up to now, different theories have been proposed to explain the origin of the catalytic efficiency of natural enzymes, and these can be used, in principle, to rationalize the consequences of point mutations in existing enzymes or to assist the process of designing new ones. The reader is addressed to different previous works to have a complete understanding of the field.^11−15^ In this respect, electrostatic interactions are recognized as the most important contribution to catalysis,^16^ in particular for the catalysis of chemical reactions where a significant electronic reorganization takes place during the limiting chemical step. According to this explanation, the enzymatic active site provides an adequate environment to stabilize, through electrostatic interactions, the reaction transition state with respect to the reactants state by electrostatic interactions more efficiently than water does when the reaction takes place in an aqueous solution.

In a chemical reaction consisting formally in the transfer of a charged group (i.e., proton, hydride, or methyl group) from a donor to an acceptor atom, the electrostatic contribution to catalysis can be approximately written as
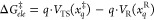
1where *V* is the electrostatic potential created on the position of the charge at the transition state (TS) or reactants state (R). Instead, if the chemical reaction involves a charge separation process, resulting in the creation of a net dipole, the electrostatic contribution can be more conveniently expressed in terms of the electric field created on that dipole:
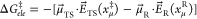
2

In general, chemical reactions may involve more complex changes in the electronic distribution on going from the R to the TS, which requires a more complete description of the electrostatic properties of the reacting system and of the active site. The free energy cost of polarizing the chemical system and the environment must also be included in a complete evaluation of the impact of electrostatic effects on the activation free energy. From a computational point of view, the calculation of electrostatic properties is usually performed using just classical (molecular mechanics, MM) nonpolarizable force fields or in combination with quantum potentials in quantum mechanics/molecular mechanics (QM/MM) schemes.^17^ In the first case, polarization is not explicitly included, while in the second case, the polarization of the quantum subsystem is included if an electrostatic embedding scheme is considered.^18^ Polarizable force fields can also be used in MM and QM/MM calculations, which introduce the polarization cost of the MM region, but these schemes are still not very popular because of the computational cost. In the case of QM/MM calculations, the differences between point charge and polarizable force fields seem to be small.^19^ Instead, current efforts are more focused in the sampling of enough configurations to get converged electrostatic descriptors and in the incorporation of long-range nature electrostatic effects using adequate schemes, such as the Ewald method.^20^

Together with the reorganization, another key element in explaining the electrostatic origin of catalysis is the concept of preorganization. The electrostatic properties of enzymatic active sites (*V* or *E⃗*) are rooted in the protein three-dimensional structure and then are largely independent of the changes taking place in the chemical system. Instead, in an aqueous solution, water molecules must be reorganized to accommodate changes in the electronic distribution of the reacting system, with the corresponding free energy penalty. In enzymes, this reorganization is minimized because the electrostatic properties of the active site are already prepared or preorganized to accommodate favorably the reaction TS. The cost of this preorganized active site in the enzyme is paid in the protein folding process.^14^

## 2 Electrostatic Potential and Enzyme Catalysis

### 2.1 Dihydrofolate Reductases (DHFRs)

**Figure 1:**
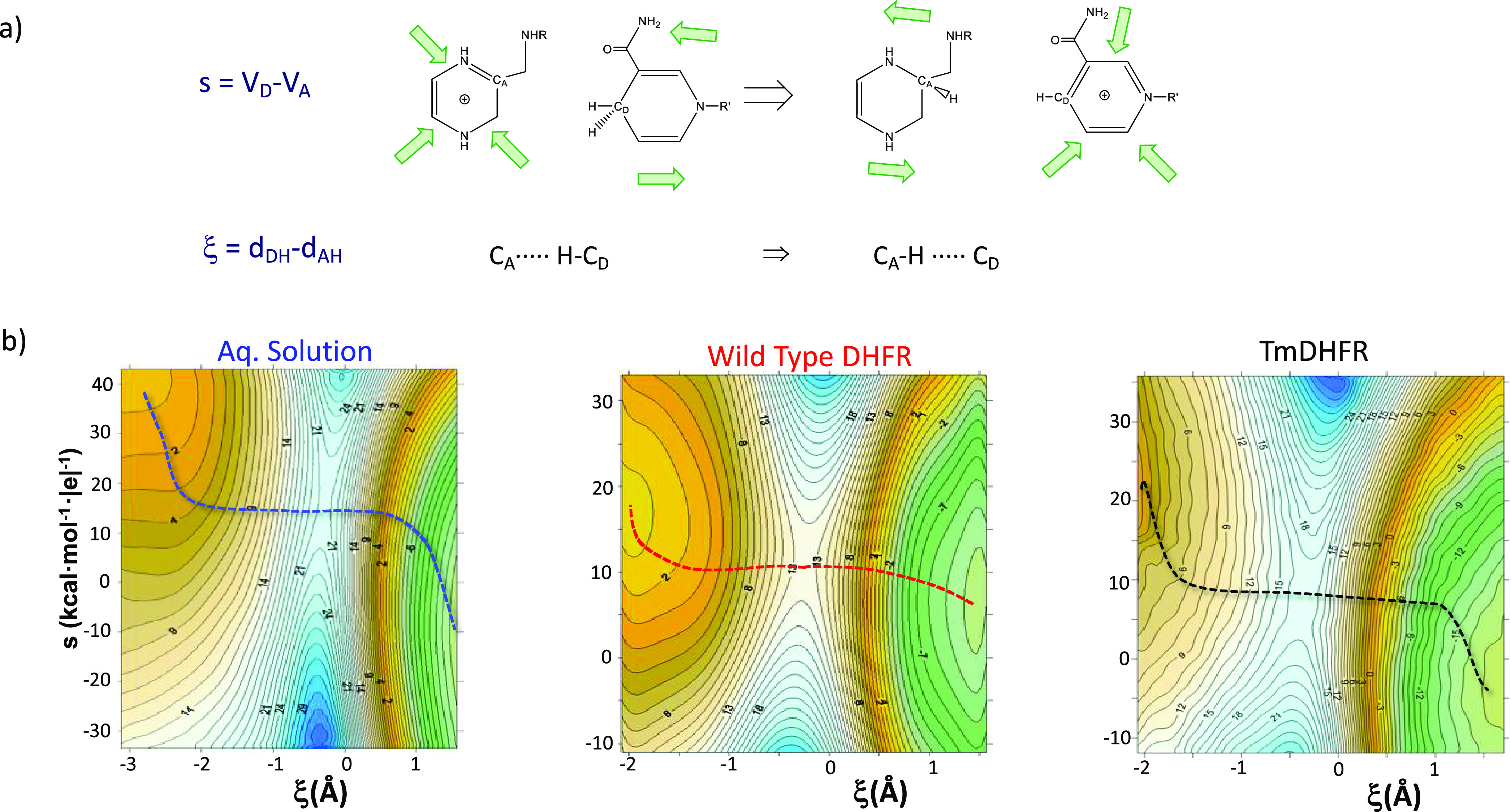
a) Hydride transfer in DHFRs and definition of a chemical and an electrostatic coordinate. The chemical coordinate is defined as the antisymmetric combination of the distances of the transferred hydride from the donor and acceptor atoms. The electrostatic coordinate, s, is the antisymmetric combination of the electrostatic potential created by the environment on the donor and acceptor atoms. b) Free energy surfaces corresponding to the hydride transfer from NADPH to protonated H_2_F in an aqueous solution (left), wild type EcDHFR (middle), and TmDHFR (right). The dotted lines represent the minimum free energy paths on the free energy surfaces obtained from the gradient of the surface. The free energy calculations were carried out at the QM/MM level, using a semiempirical description of the QM subsystem with specific reaction parameters. The figure is adapted from ref (23). See ref (23) for the computational details.

Simulations offer the opportunity to dissect the full catalytic effect observed in experiments into different contributions, isolating those contributing the most to the experimental observation. This is also the case for the electrostatic contributions to catalysis in enzymes. Dihydrofolate Reductases (DHFRs) are a paradigmatic example to analyze the origin of catalytic effects.^21−23^ DHFR catalyzes the transfer of the pro-R hydride from the C4 position of NADPH and a proton from water to the C6 and N5 positions of dihydrofolate, respectively, with the hydride transfer being the slower step in the chemical process. The free energy landscape of the reaction can be explored in terms of a chemical coordinate that defines the transfer of the hydride from the donor carbon atom to the acceptor carbon atom (see Figure 1a). However, a more complete perspective can be obtained by adding an environmental coordinate that measures the electrostatic effects on the hydride transfer. This can be accomplished with the antisymmetric combination of the electrostatic potential created by the surroundings on the donor and acceptor atoms, reflecting the ability of the environment to stabilize a charge on one site or the other. A positive value of the electrostatic environmental coordinate, denoted as s in Figure 1, indicates an electrostatic potential favoring the positioning of the hydride on the donor side, while a negative value indicates an environment favoring the hydride on the acceptor site. Figure 1b shows the free energy landscapes for the uncatalyzed reaction (in an aqueous solution) and in two DHFRs: *E. coli* DHFR (EcDHFR) and *Thermotoga maritima* DHFR (TmDHFR, an enzyme adapted to work at high temperature).^24^ At room temperature, EcDHFR is significantly more efficient than TmDHFR. The free energy landscapes illustrate the role of electrostatic effects on catalysis. The chemical reaction involves changes in both the chemical and the environmental coordinates, with the surroundings being adjusted to stabilize the reaction TS. The sequential nature of these changes reflects the different characteristic times of these coordinates: changes in the environment are slower than the fast hydride transfer. The enzymes provide a much better preorganized environment at the reactant state, presenting values of the environmental coordinate significantly closer to that reached at the TS than water does. Conversely, in all cases, we see a reorganization of the environment as the reaction advances: the environmental electrostatic coordinate s changes from the reactant state to the TS by about 25, 14, and 8 kcal·mol^–1^·|e|^–1^ in solution, TmDHFR and EcDHFR, respectively. The magnitude of this reorganization is inversely correlated to the efficiency of the environment to promote the reaction (EcDHFR > TmDHFR > aqueous solution). This analysis of the catalytic efficiency in terms of electrostatic preorganization can also be used to dissect the effects of mutations. The reduced catalytic efficiency in the N23PP/S148A variant, initially assigned to a “dynamical knockout”,^22^ can be explained as a loss in the degree of preorganization of the active center as a result of mutations.^23^

This electrostatic picture of catalysis provides insightful lessons for the design of new biocatalysts. First, the design must consider the electrostatic properties of the TS, which must be stabilized by the environment with respect to the reactant complex. Second, one should also pay attention to protein flexibility to avoid the free energy prize associated with a large reorganization when moving from reactants to the TS: that is, the environment must be already sufficiently preorganized at the reactants state.

### 2.2 Glycine *N*-Methyltransferase (GNMT)

**Figure 2:**
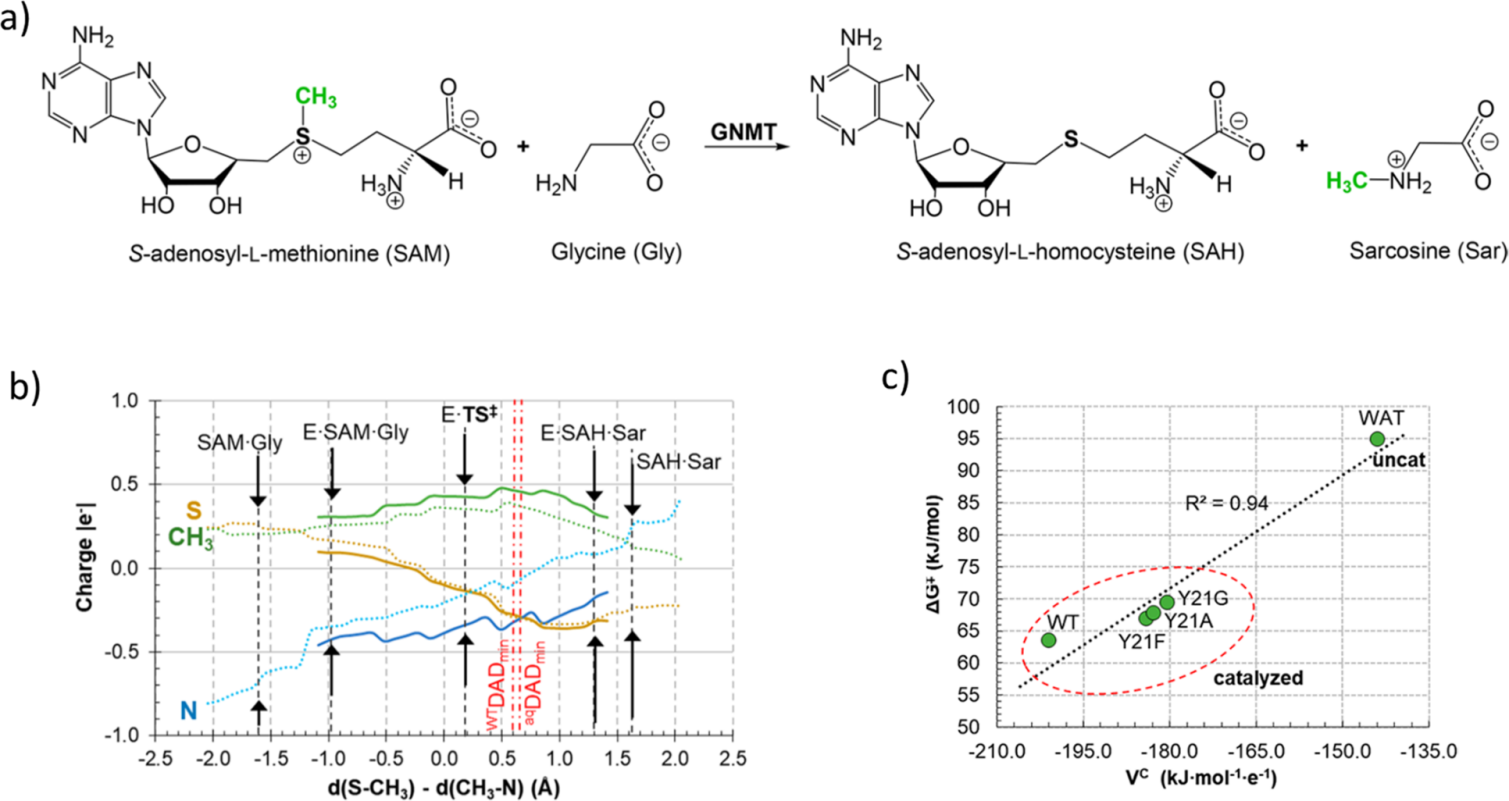
a) Chemical step of the GNMT catalyzed reaction. b) Evolution of charges (in au) of the donor atom, S, acceptor atom, N, and the sum of the charges of the transferring methyl group, CH_3_, along the reaction path. c) Relationship between activation free energies and the averaged electrostatic potential created by the environment (protein or water molecules) on the carbon atom of the transferring methyl group at the TS structures (R^2^ = 0.94 for linear regression). The figure is adapted from ref (26).

GNMT is an *S*-adenosyl-l-methionine (SAM)-dependent enzyme that catalyzes the transformation of glycine into sarcosine by means of an S_N_2 methyl transfer reaction (Figure 2a). As in other enzyme catalyzed S_N_2 reactions, it has been suggested that specific protein fluctuations might reduce the donor–acceptor distance (DAD), thus diminishing the potential-energy barrier height and/or width and enhancing the rate by increasing the number of reactive trajectories over and through the barrier. The reduction of the catalytic efficiency of GNMT when active-site residue Y21 was replaced by a series of mutants, as well as the change in the secondary α-^3^H Kinetic Isotope Effects (KIEs), was interpreted a support of this DAD “compression” effect.^25^ However, activation free energies derived from computed free energy surfaces (FESs) and KIEs of wild-type GNMT and 3 variants (Y21F, Y21A, and Y21G) by means of Variational Transition State Theory (VTST) showed that the mutations do not meaningfully affect the DAD.^26^ In particular, the FESs were computed as 2D potential of mean force (PMFs) at the AM1/MM level followed by corrections of the QM region with DFT functionals (M06-2X) and optimization of TSs at the DFT/MM level to confirm the topology of the corrected surfaces. On the contrary, electrostatic properties in the active site of the four studied enzymes correlate with their catalytic activities. QM/MM simulations showed how the methyl-group charge reaches a maximum close to the TS and decreases again in the product state (Figure 2b). This is related to the evolution of the averaged electrostatic potential created by the different environments at the S donor, N acceptor, and C methyl atoms. The wild type (WT) enzyme is the protein that generates the most favorable electrostatic environment to stabilize the charge developed on the methyl group in the TS, and thus, it is the most favorable environment to catalyze the reaction. The knowledge obtained from the analysis of the reaction mechanism and, in particular, the evolution of the charges on the reactive system from reactants to products guides the selection of the adequate electrostatic properties to be correlated with the enzymatic efficiency. Indeed, an almost perfectly lineal relationship between activation free energies and the averaged electrostatic potential created by the environment on the carbon atom of the transferring methyl group at the TS structures were computed (Figure 2c). The detrimental effect of substitution of Y21 on the electrostatic potential is partially compensated by H142 that, by approaching the methyl group, generates a higher potential. In this case, the electrostatic potential calculated at the TS on the carbon atom is an excellent predictor of the activity of the different enzyme variants not only because at this stage the methyl group presents the maximum positive charge but also because at the TS the positive charge is more localized (see Figure 2b). Exactly the opposite of what happens in a typical S_N_2 reaction, where the negative charge is more localized in the reactants than in the transition state.

It must also be taken into account that the changes on the electrostatic potential exerted by the different proteins on the substrate reflect that they are not static but dynamic macromolecules that have to evolve structurally from the reactant state to the TS, although at a low energy cost. In fact, the plasticity of the WT protein is responsible for the differences in the KIEs and rate constants after mutations being as small as they are, in contrast to the changes from the reactant state to the TS that are significantly more dramatic in water.

### 2.3 20S Proteasome

**Figure 3:**
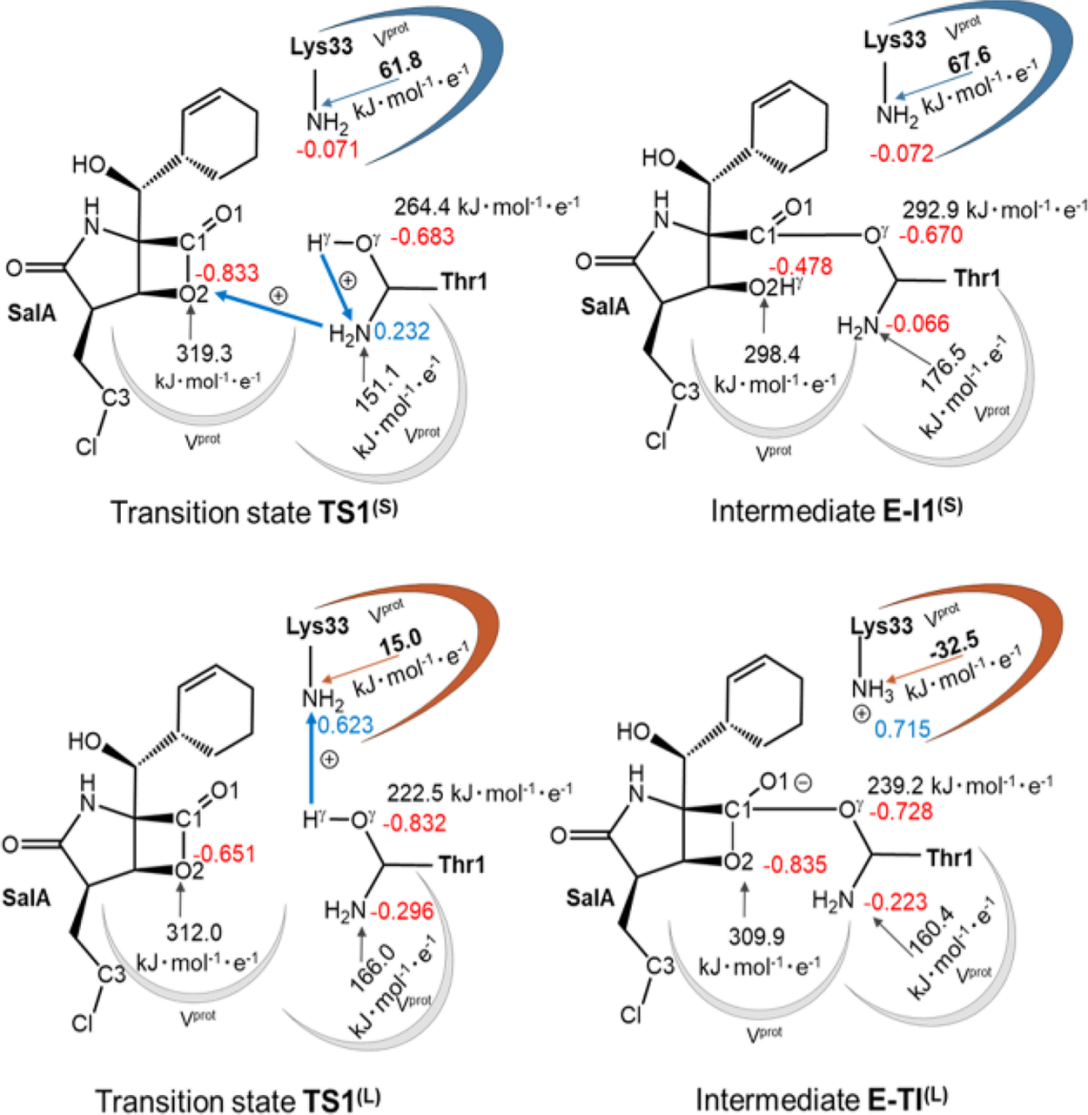
Averaged values of the electrostatic potential (V^prot^, in black), charges (in red and blue), and schematic representation of the flow of charges taking place in the states appearing along the first step of the inhibition of 20S proteasome with SalA in both SalA-assisted (S) and Lys-assisted (L) mechanisms are illustrated as blue arrows in the TSs. This figure is adapted from ref (27).

20S proteasome is the enzymatic core engine involved in the processes of cellular protein regulation, thus becoming a promising target inter alia in the therapy of many diseases. The origin of the activity and the inhibition mechanism of 20S proteasome has been elucidated based on the generation of the free energy landscape computed (as in a previous case, with AM1/MM 2D PMFs followed by corrections of the QM region with a DFT functional and optimization of TSs at the DFT/MM level to confirm the position of the quadratic region on the corrected surfaces) and analysis of the electrostatic effects.^27^ Based on crystallographic and kinetic studies, the inhibition of 20S proteasome (with different kinds of compounds) has been proposed to involve the formation of the covalent ester bond between Thr1 and the inhibitor (Figure 3). However, the question is how the catalytic Thr1 residue is activated. A computational study of the inhibition of the β5 subunit target of drugs accepted by the FDA, by a nonpeptidic β-lactone-γ-lactam compound (salinosporamide A, SalA), showed the existence of a favorable pathway, different from the widely accepted SalA-assisted mechanism.^28^ Analysis of the electrostatic features of 20S proteasome (Figure 3) revealed the importance of the electrostatic preorganization/reorganization of the enzyme and the pivotal role of Asp17 in modulating p*K*_a_ of Lys33 and explains how a molecule, completely unlike the natural substrate of 20S proteasome, binds and inhibits its active site. These results are an example of how the detailed acknowledgment of the electrostatic effects of enzymes can be used not only to understand the origin of enzyme catalysis but also to refine efficient inhibitors, with consequent potential applications in medical treatments.

## 3 Electric Fields in Enzymatic Reactions

### 3.1 Catechol *O*-Methyl Transferase (COMT)

**Figure 4:**
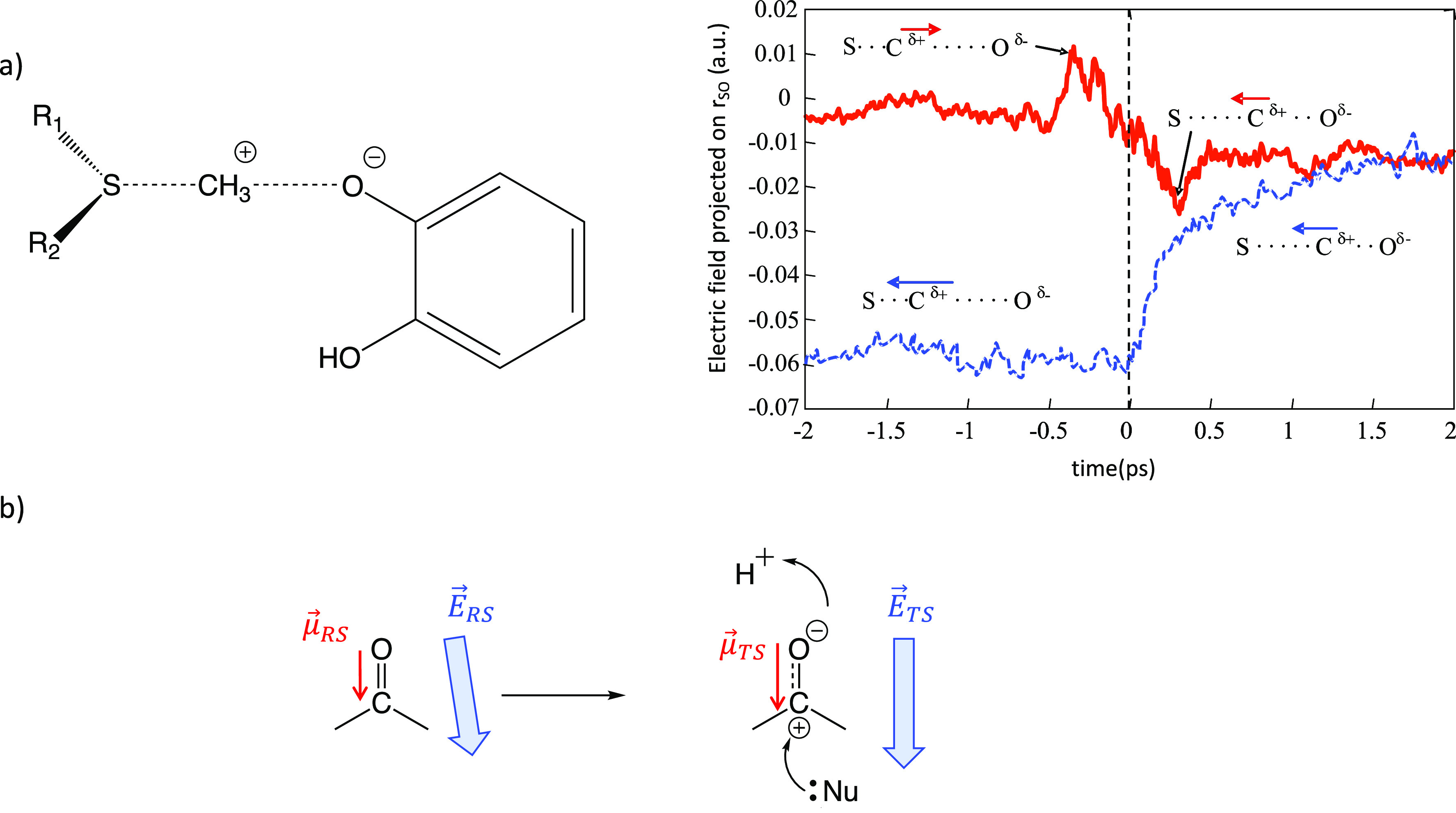
Illustration of electric field effects on two enzymatic reactions. a) Reaction catalyzed by Catechol *O*-Methyl Transferase and projection of the electric field on the methyl group along the donor–acceptor axis in an aqueous solution (blue line) and in the enzyme (red line). The electric field was obtained as the average of rare event simulations started at the reaction TS (*t* = 0) using a QM/MM approach. b) Polarization of carbonyl groups in enzymatic reactions and the effect of the electric field favoring the stabilization of the transition state. This figure is adapted from ref (30). See ref (30) for details.

In some cases, electrostatic effects on chemical reactivity can be better rationalized in terms of the electric field and the potential gradient. This is particularly true when a chemical reaction involves a substantial change in the orientation or magnitude of a dipole moment.^29^ Then, optimizing the positions of the partial charges under the effect of the electrostatic potential can be explained in terms of the dipole orientation with an electric field. One paradigmatic example is the reaction catalyzed by Catechol *O*-Methyl Transferase (COMT), where a positively charged methyl group is transferred to the negatively charaged oxygen atom of the substrate, catecholate. In this reaction (see Figure 4a), a large dipole moment is annihilated as the reaction proceeds. The electric field calculated on this methyl group in the donor–acceptor direction clearly shows the differences between the uncatalyzed reaction in an aqueous solution and the catalytic process.^30^ In an aqueous solution, the electric field created by the solvent is a reaction field opposed to the reaction progress: the charged reactants interact more strongly with the solvent than the products. Instead, the electric field in the enzymatic active site is already preorganized in the Michaelis complex and changes substantially less than it does in an aqueous solution. In addition, after a small reorganization, due to the reorientation of the substrate with respect to a magnesium ion present in the active site, the force due to the electric field (*F⃗*_*ele*_*= −q·E⃗*_*TS*_) pushes the methyl group toward the TS configuration (see Figure 4a). This reaction clearly illustrates that while the reaction field in an aqueous solution depends on the solute’s charge distribution, an enzyme, due to its structure, can create a preorganized electric field, with a given orientation and magnitude, such that favors the chemical reaction.

Experimentally, the effect of the electric field can be monitored through its impact on the vibrational properties due to the Stark effect. Vibrational Stark spectroscopy records the effect of an external electric field on the infrared spectrum of a molecule.^31^ Because of the different dipole moments between two vibrational levels, their energy is affected differently by the applied electric field resulting in a shift of the signal frequency that can be approximated as^32^
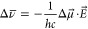
3

The sensitivity of a molecular vibration to the electric field can be calibrated by measuring the frequency shifts under different known electric fields and using the obtained calibration to find out the value of the electric field in a particular environment once the infrared spectrum has been recorded. A good probe for this effect is the infrared vibrational signal associated with carbonyl groups, what has been shown to keep a linear dependence on electric fields in different environments.^33^ The measurement of the electric field acting on a carbonyl bond can be relevant for understanding catalytic effects because the local dipole moment of this group is increased in many enzymatic catalytic reactions (Figure 4b). If the dipole moment at the TS is larger than at the reactants state, the electric field created by the enzyme, if correctly oriented, can reduce the activation free energy and then increase the reaction rate constant. This is what Boxer and co-workers observed in the case of Ketosteroid Isomerase (KSI), where the electric field determined by means of the Stark effect at the active site of a series of KSI mutants correlated linearly with the activation free energy.^33,34^ This enzyme catalyzes the transformation of a steroid substrate via enolization and reketonization of the carbonyl group of the substrate. Mutations and chemical modifications that decrease the magnitude of the electric field along this carbonyl group result in reduced catalytic activity. Beyond enzyme catalysis, the role of electric fields in chemical reactivity has also been evidenced for heterogeneous catalysis of a Diels–Alder reaction. The theoretical prediction that electric fields could lower the activation barrier of this process was evidenced using single-molecule scanning tunnelling microscopy which provided an electric field properly oriented to catalyze the reaction between the diene and the dienophile taking place on a surface.^35,36^

### 3.2 Breaking and Forming of the Peptide Bond

**Figure 5:**
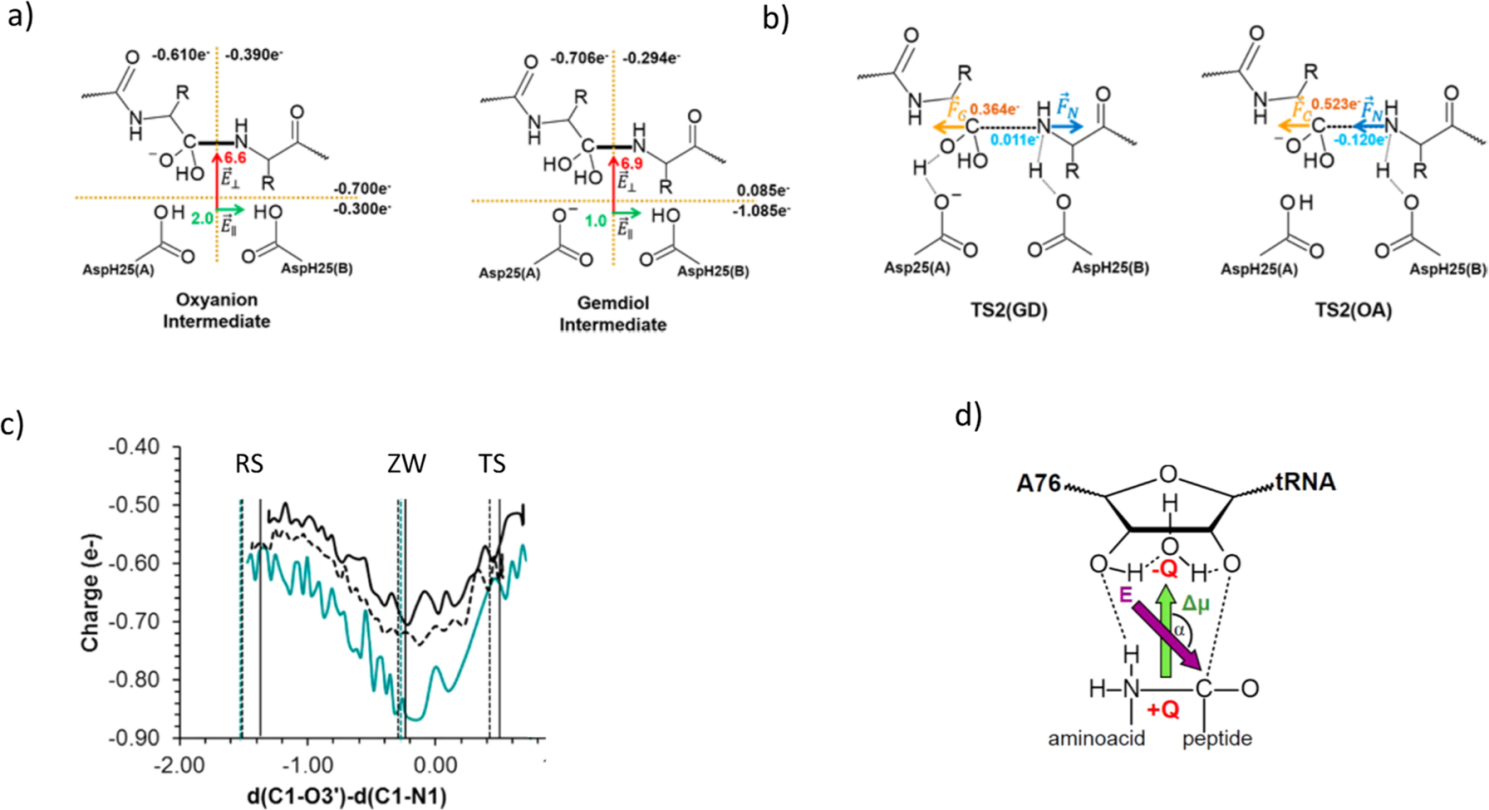
a) Electronic charge (in au) summed into the different fragments separated by two imaginary planes represented by dashed lines, projection of the electric field (in au ×10^3^) created by the protein and water molecules in the C–N peptide bond direction (green arrows) and in a perpendicular direction (red arrows), computed in the center of the active site. b) Atomic charges computed for structures of the rate-limiting step of the gem-diol mechanism, TS2(GD), and the oxyanion hole mechanism, TS2(OA), and schematic representation of the electrostatic forces created by the protein on the C and N atoms, projected on the C–N scissile peptide bond direction. c) Evolution of the atomic charge on oxygen O1 for the 8-membered ring mechanism in water (cyan line) and Ribosome (black lines) (The solid line corresponds to the Ribosome with additional Mg^2+^ ions.). Vertical lines indicate the position of the reactant state (RC), zwitterion (ZW), and transition state (TS) in the different environments. d) Alignment of the electric field created by the environment with the dipole moment appearing during the chemical reaction. The figure is adapted from refs (37 and 38).

The peptide bond is the vital link that connects amino acids to form proteins in living organisms. Thus, enzymes have evolved over millions of years to catalyze either the forming or the breaking process for different purposes, and apparently, electrostatic effects appear to be crucial in the catalysis of the two inverse reactions. HIV-1 Protease (HIV-1 PR) is one of the three enzymes essential for the replication process of the HIV-1 virus, which explains why it has been the main target for the design of drugs against acquired immunodeficiency syndrome (AIDS). Despite the relatively simple structure of the active site (Figure 5a) and the huge number of experimental and computational studies, the molecular mechanism and the origin of catalysis is still a question of debate. Generally, there is enough evidence that a water molecule is activated by an aspartate residue, and it then attacks the carbonyl carbon of the substrate peptide chain. Nevertheless, different mechanisms for the reaction catalyzed by aspartic proteases have been suggested, including a concerted mechanism, a mechanism via an oxyanion intermediate, and a mechanism via a gem-diol intermediate.

QM/MM FESs (computed, as in previous cases, with AM1/MM 2D PMFs followed by corrections of the QM region with a DFT functional and optimization of TSs at the DFT/MM level to confirm the position of the quadratic region on the corrected surfaces) suggested that the most favorable reaction mechanism is the one involving formation of a gem-diol intermediate, whose decomposition into the product complex would correspond to the rate-limiting step.^37^ The agreement between the activation free energy of this step with experimental data as well as KIEs supports this prediction. The role of the protein dynamics was studied by protein isotope labeling in the framework of the VTST. The predicted enzyme KIEs, very close to the values measured experimentally, reveal a measurable but small dynamic effect. Calculations showed how the contribution of dynamic effects to the effective activation free energy appears to be below 1 kcal·mol^–1^. On the contrary, the electric field created by the protein in the active site of the enzyme emerges as being critical for the electronic reorganization required during the reaction. Thus, the electrostatic effects of HIV-1 PR were estimated by analyzing the evolution of charge transfer in the active site of the protein and by computing the electric field generated by the enzyme in the center of the active site. Analysis of the electric field, projected in two directions defined by the scissile C–N bond and the one perpendicular to this bond into the direction of the two active site aspartate residues, suggested that the transfer of a negative charge from the aspartate residues to the region where the peptide is located represents a nonfavorable process for the enzyme, while the transfer along the scissile peptide bond does not require a significant energetic penalty (Figure 5b). The value of the projection of the electric field in the direction of the scissile C–N bond is small, which means that the electric work to move a charge in this direction is negligible, while a positive value of the electric field in the perpendicular direction, from bottom to top, means that an electric work would be required if a negative charge would be displaced in this direction. In fact, the decomposition of the electrostatic forces generated by the protein in the scissile peptide bond on the rate limiting transition state would favor the peptide bond cleavage. Thus, it appears that electrostatic effects on HIV-1 PR favor the peptide bond breaking process.

In contrast, the peptide bond formation reaction is catalyzed in living organisms by the ribosome, considered as an ancient enzyme (ribozymes), responsible for the flow of the genetic information encoded within genes into proteins. This is an excellent example to test the relevance of electrostatic effects in catalysis since the system evolves from two neutral species in the reactants to the formation of an ion pair-like TS through a zwitterion-like intermediate. In a previous study, after exploring the mechanism in solution and in the ribosome, the electrostatic coupling between the chemical subsystem and the environment was monitored in both media.^38^ Analysis of the reaction through the most favorable reaction path in both media confirmed the larger electron density reorganization along the process in solution than in the enzyme (Figure 5c). The charge separation of the solute that takes place during the reaction is stabilized and amplified in solution but pays an entropic penalty. In contrast, the ribosome saves this entropic cost, offering a more rigid environment that is preorganized at the Michaelis complex. Consequently, the charge separation of the solute during the reaction is dampened in the ribosome, making it less polar. However, the dipole moment on the TS of the reacting subsystem is stabilized by a properly oriented electric field created by the environment (Figure 5d). This observation is in agreement with the enthalpic and entropic differences experimentally measured. An intriguing aspect of the ribosome, by comparison with protein enzymes, is that the full catalytic effect seems to be already attained at the Michaelis complex.

## 4 Electrostatically Guided Enzyme Design

Despite the emerging interest in designing new enzymes to solve practical challenges, the use of computer-based approaches to redesign catalytically active proteins remains largely unexplored, in particular, in the case of those approaches based on electrostatic principles. Once the relevance of electrostatic effects in enzyme catalysis is demonstrated, more specifically in the relative stabilization of the TS with respect to the reactants state (reflected in a reduction of the activation free energy), the next challenging aim would be to design new catalytic environments based on this premise. Some examples are summarized below. These examples have been selected because of their historical (Kemp eliminase) and practical importance (amidases and PETases) and also because, until now, they are some of the few examples where the computation of electrostatic properties has been demonstrated to be a powerful tool to rationalize the design process and that may be useful to guide the design of new biocatalysts.

### 4.1 Kemp Eliminase (KE)

**Figure 6:**
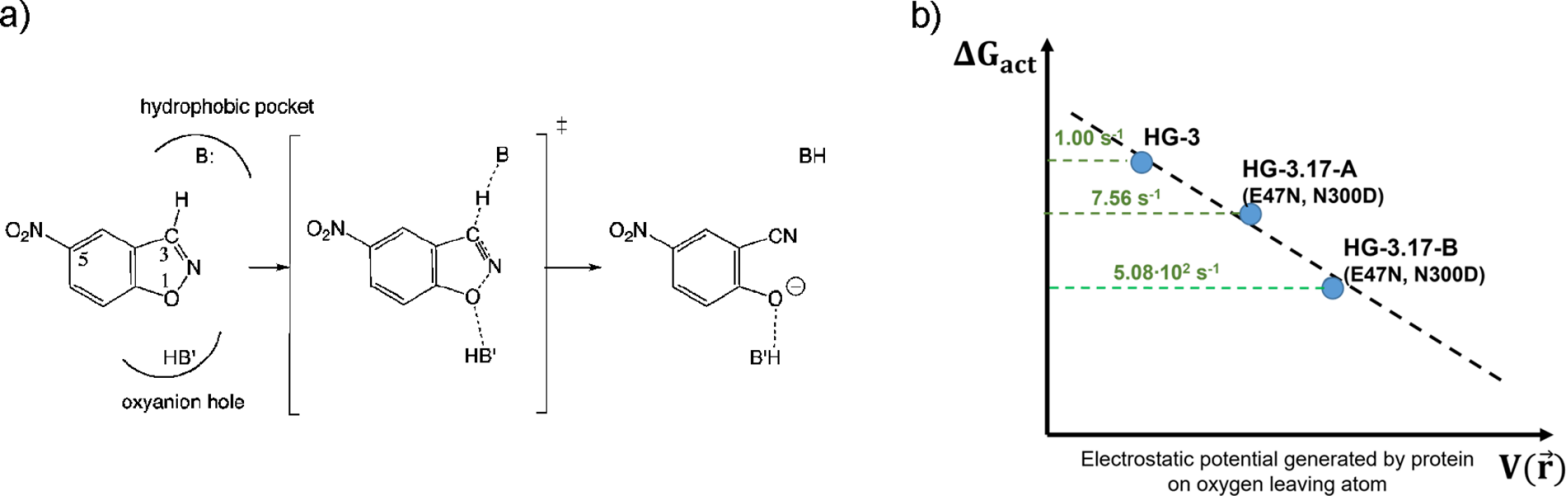
a) Schematic representation of the base-catalyzed Kemp elimination of 5-nitrobenzisoxazole. b) The correlation between the activation free energies (derived from FESs computed by means of the umbrella sampling approach with QM/MM MD simulations) and the electrostatic potential generated by the proteins on the oxygen leaving group is shown as blue dots. Theoretical predictions (from refs (42 and 43)) are shown as blue dots, while experimental kinetic data are shown in green numbers.

The KE reaction, which consists of the conversion of benzisoxazoles into salicylonitriles (Figure 6a), is an interesting reaction to analyze the electrostatic potential effects in catalysis because it implies a proton transfer from a carbon atom to a heteroatom. In addition, since no naturally occurring enzymes have been identified to catalyze this reaction, it has been used as a benchmark of different protocols to design new enzymes. Several remarkable achievements have been published since the first *de novo* design by Houk, Tawfik, Baker, and co-workers,^39^ in which special attention was focused in stabilizing the developing negative charge on the phenolic oxygen atom. After this first *de novo* protein design, an iterative approach starting from an inactive protein, HG-1, was used to convert the xylan binding pocket of a thermostable xylanase into a KE, focused in the geometry and solvent access of the active site and the flexibility of the too flexible character of the protein.^40^ The newly designed enzyme, HG-3, catalyzed the KE of 5-nitrobenzisoxazole with a rate constant *k*_cat_ = 0.68 s^–1^. Starting from this computational designed catalyst, a highly active KE was later redesigned by Hilvert and co-workers by means of an evolutionary strategy that included both global and local mutagenesis.^41^ The most active variant, HG-3.17, showed an efficiency close to that of natural enzymes (*k*_cat_ = 700 ± 60 s^–1^). The improvement of the KE from HG3 to HG-3.17 was attributed not only to the extraordinary high shape complementarity between the binding pocket of the protein and the substrate but also to bond interactions with base D127 and the stabilization of the negative charge on the O1 atom of the substrate at the transition state.

A deep analysis of the base-catalyzed KE of 5-nitrobenzisoxazole catalyzed by HG3 and HG-3.17 was carried in our laboratory based on results derived from computational techniques.^42,43^ Our studies showed that the high reactivity of HG-3.17 was related to a proper electrostatic preorganization of the environment that creates a favorable electrostatic potential for the reaction to proceed, especially on the oxygen leaving group that is negatively charged as the reaction proceeds. QM/MM molecular dynamics (MD) simulations allowed identifying the presence of different conformations in HG-3.17 with significantly different reactivity,^42^ being the larger reactivity related with a better electrostatic preorganization of the environment. When including the HG-3 KE into the comparison,^43^ the conclusions were supported, obtaining a linear correlation between the activation free energies and the electrostatic potential generated by the protein in the leaving group (Figure 6b).

Head-Gordon and co-workers arrived at similar conclusions on the relevance of the electrostatic preorganization in the efficient designs of previous KEases and suggested that efficient computationally designed enzymes could be achieved with minimal experimental intervention using electric field optimization as guidance.^44−46^ In this case, authors improved the efficiency of a *de novo* designed Kemp Eliminase enzyme (KE15) (from a value of *k*_cat_/K_M_ of 27 to 403 M^–1^ s^–1^), by predicting (and later tested experimentally) 4 mutations that enhanced the electric fields and chemical positioning of the substrate to stabilize the transition state.

A reaction mechanism of the KE catalyzed reactions different from the traditional acid/base mechanism (Figure 6a) is a redox mechanism assisted by an active site heme group.^47^ An example of an enzyme employing this mechanism for the KE reaction is the P450-BM3 mutant of Shaik, Reetz, and co-workers, which showed a 10^7^-fold larger rate constant over the uncatalyzed process in solution.^48,49^ Other heme-containing proteins have been shown to catalyze the KE, such as several aldoxime dehydratases (Oxds).^50^ It has been postulated that the ferrous heme group, and not the ferric heme, is the one that is coordinated to the substrate aldoxime and catalyzes the process, providing an electron to the substrate during the first step and receiving it back in the second one. An interesting question is whether the secondary KE reaction catalyzed by OxdA follows the same mechanism as the primary reaction, as previously assumed.^50^ An adequate electrostatic environment to stabilize the negative charge developed in the oxygen leaving group was also observed as crucial in the catalysis of the KE reaction catalyzed by a heme-dependent promiscuous aldoxime dehydratase (OxdA).^51^

### 4.2 Transforming an Esterase into an Amidase

**Figure 7:**
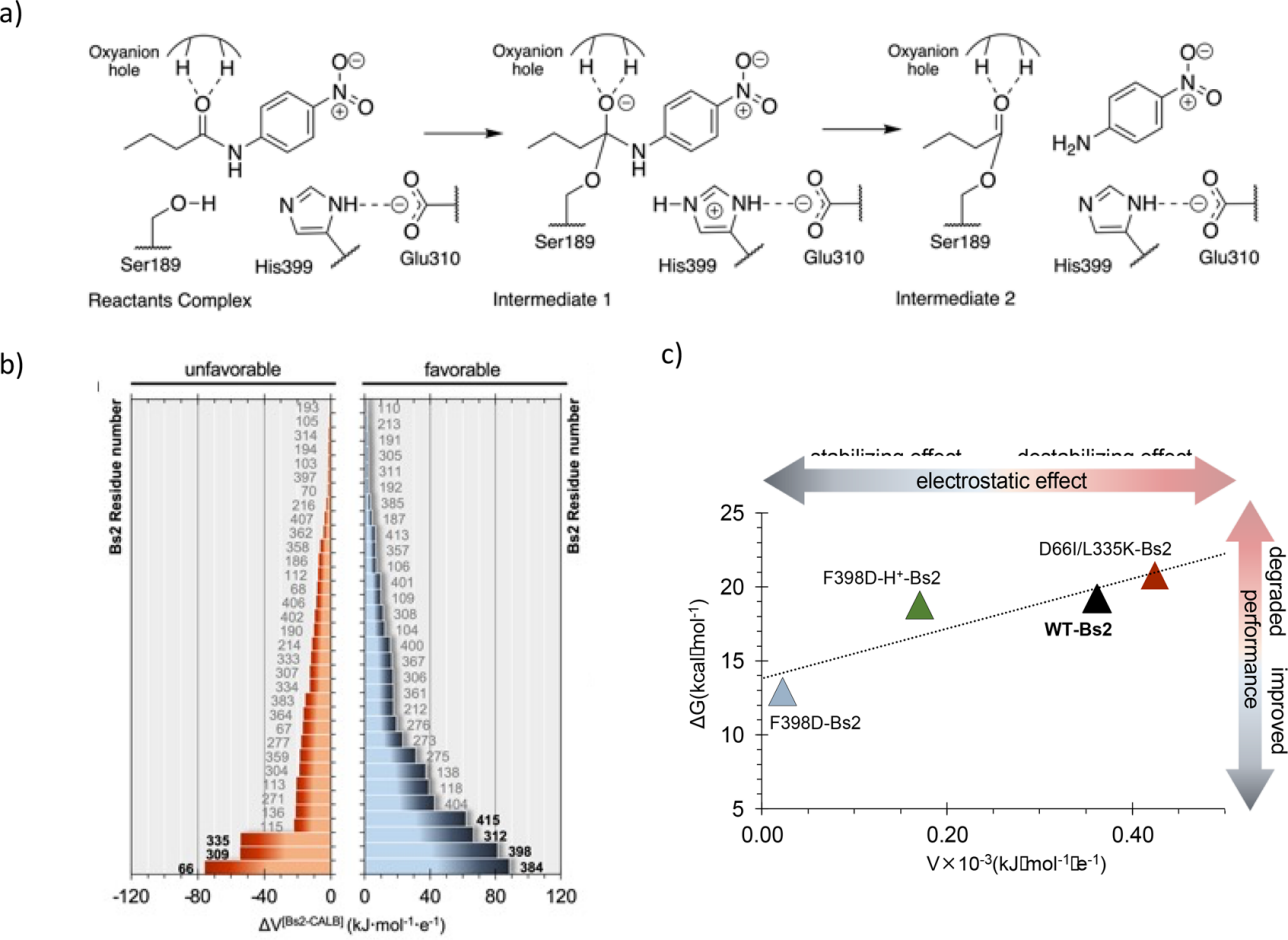
a) Acylation step of the amidase reaction catalyzed by Bs2 and CALB. b) Difference in the electrostatic potential (ΔV) generated on the catalytic histidine in the TS1 structure by each residue of Bs2 and CALB, calculated as Vi(Bs2) – Vj(CALB) where *i* and *j* are the corresponding paired residues of each enzyme. c) Correlation between the activation free energy of the acylation step (derived from FESs computed by means of the umbrella sampling approach with AM1/MM MD simulations followed by DFT/MM corrections) and the electrostatic potential in the Nε atom of His. This figure is adapted from ref (52) (Copyright 2022 Royal Society of Chemistry).

The use of promiscuous enzymes to redesign improved variants is one of the most promising strategies nowadays. We recently proposed a rational QM/MM MD strategy based on the combination of the best electrostatic properties of different promiscuous enzymes with activity on a common reaction.^52^ The proof of concept was applied to the redesign of an existing promiscuous esterase to enhance its secondary amidase activity. By focusing on two promiscuous esterases, *Candida antarctica* lipase B (CALB) and the esterase from *Bacillus subtilis* (Bs2), the first step of the protocol was to carry out an in-depth analysis on the electrostatic properties of the chemical transformations under the effect of the protein along the reaction, explored by QM/MM MD simulations (Figure 7a). The results illustrated that the rate-limiting transition states (step 1 of the acylation) could be further stabilized by generating a negative electrostatic potential on the chemical system, where a positive charge was developed (active site histidine residue) through site-directed mutagenesis. Then, the next question was to determine which esterase should be selected as the protein scaffold to perform the mutations. To answer this question, an evolutive analysis of the protein geometries using the Convolutional Neural Network (CNN) deep learning approach was allowed to predict the classification of both enzymes and confirmed their significant structural differences.^53^ According to the results, Bs2 would be a more robust protein scaffold to perform mutagenesis studies in order to improve amidase activity without dramatically perturbing the structure of the protein. On the contrary, mutations on CALB appeared to have significant effects on the 3D structure of the protein. The alignment of the structures of both systems from the trajectories in the first transition state of the amidase reaction was done by using a rotation quaternion. Key residues with a different impact for the amidase reaction in the two enzymes were identified; residues of the Bs2 with an unfavorable effect on catalysis were substituted by those at equivalent spatial positions in CALB that exert a favorable effect (Figure 7b). Our *in silico* method predicted an, *a priori*, increase of 4 orders of magnitude on the measurable rate constant after a single mutation of Phe398 to aspartate, which is ∼10 Å away from the active site. However, deeper computational insights revealed a significant shift in the p*K*_a_ of the inserted aspartate, resulting in a more modest catalytic effect. This prediction was experimentally confirmed as a 1.3-fold increase in activity which can be further improved with mutations. While a variant with much better activity was not obtained, there was excellent agreement between the predicted activation free energies and the experimentally determined rate constants. In addition, the same strategy was employed to prepare variants of Bs2 with lower catalytic activities as a control test of the method, fulfilling the correlation between the electrostatic potential on the histidine residue exerted by the protein and the activation free energy (Figure 7c).

Supported by the good agreement between theoretical and experimental results, as well as the linear correlation between the electrostatic properties and the activation energy barriers (Figure 7c), the presented computer-guided rational design can become a valuable component in the toolkit of enzyme redesign and engineering, upon optimization.^52^

### 4.3 Improving the Design of PETases Using Electrostatics

Biocatalysts have emerged as a promising solution for the treatment of plastic waste, one of the most pressing environmental problems. The finding that some enzymes can decompose polymers has paved the way for a possible bioremediation of the contamination caused by plastics and for the upcycling of the products resulting from their hydrolysis. In particular, the performance of biocatalytic degradation of polyester-type plastics, such as polyethylene terephthalate (PET), has been significantly enhanced in recent years.^54^*Ideonella sakaiensis* PETase (IsPETase) is an interesting target due to its capability of degrading PET at room temperatures^55^ and has received considerable attention as a starting point for the bioremediation of PET contamination. Improving the biocatalytic decomposition of PET is undoubtedly a multifaceted problem that requires improving several factors such as the accessibility of plastic fibers to the enzyme, the affinity between the enzyme and the plastic chain, the thermostability of the proteins, and its catalytic efficiency.^56^

**Figure 8:**
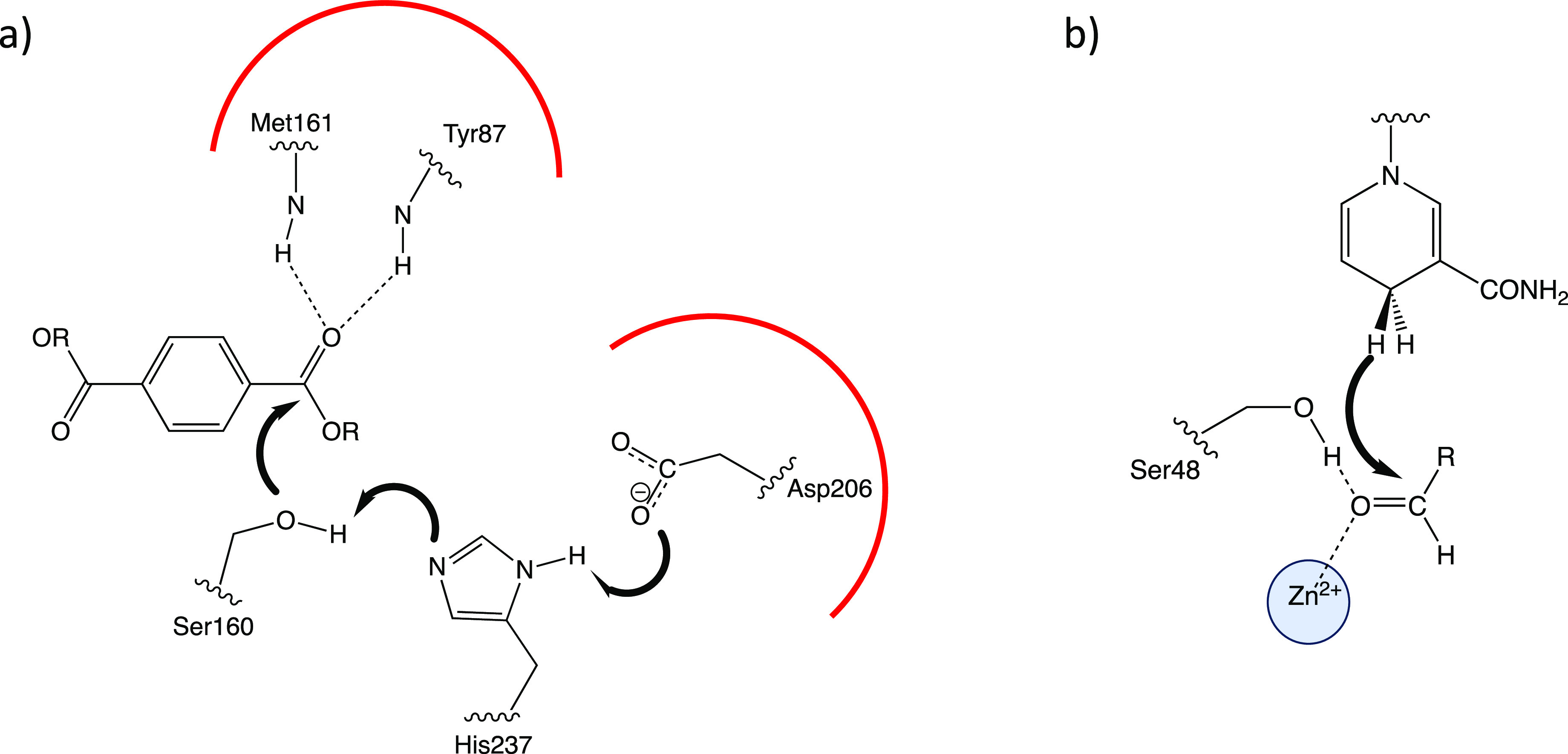
a) Active site of IsPETase indicating the two regions where electrostatic interactions can be enhanced to improve catalysis. b) Active site of horse liver alcohol dehydrogenase showing important electrostatic interactions with the substrate’s carbonyl group.

The active site of IsPETase contains a typical catalytic triad (Ser160-His237-Asp206), and two amino acids (Met161 and Tyr87), able to interact with the oxygen atom of the PET carbonyl group though H-bonds (oxyanion hole), as shown in Figure 8a. The reaction mechanism involves two steps: acylation and deacylation. According to free energy simulations carried out at the QM/MM level with different QM descriptions,^57,58^ during the first step, the activation of the nucleophilic Ser160 is carried out by His237, whose p*K*_a_ is modulated by Asp206. Once activated, the side chain oxygen of Ser160 attacks the electrophilic carbon atom of the polymer ester bond. The negative charge developed on this last atom can be stabilized by the oxyanion hole. Structural analysis of the evolution of the active site along the reaction progress and the study of electrostatic effects generated by the IsPETase, in comparison with that of the improved ICCG variant metagenome-derived leaf-branch compost cutinase (LCC-ICCG), reveal a similarity in the behavior of the active site of these two enzymes. These results suggested that the origin of the apparent better performance of the LCC-ICCG protein over IsPETase must be due to its capabilities of working at higher temperature and its intrinsic relationship with the crystallinity grade of the polymer.^57^

Several mutants have been proposed to increase the activity or thermal stability of IsPETase. Particularly relevant is the case of the FAST-PETase enzyme, designed by machine learning techniques that identify stabilizing mutations. This variant contains only five mutations with respect to wild-type PETase and presents better catalytic activity than the wild-type PETase and other proposed mutants.^59^ Interestingly, FAST-PETase displays improved properties for the degradation of untreated PET waste. A computational analysis based on the calculation of the minimum free energy path at the QM/MM level showed that the reason for the improved catalytic activity is of an electrostatic nature. The FAST-PETase presents an environment around Asp206 that enhances its basic character with respect to the wild-type enzyme, reducing the energy cost associated with the proton transfer from the nucleophilic serine (Ser160) to the histidine His237.^58^

Another strategy to improve the catalytic efficiency of PETases from an electrostatic perspective focuses on the increased dipole of the carbonyl group when the reaction TS is reached, as shown in Figure 4b. A recent study proposed a new computational rational design of an enzyme for mono(2-hydroxyethyl) terephthalate (MHET) hydrolysis, an intermediate of PET depolymerization that can inhibit the enzymatic activity of PETases.^60^ The guiding principles to design the active site were the binding of the reaction TS in a catalytically active conformation, the presence of the catalytic triad, and the strengthening of the oxyanion hole hydrogen bonds that stabilize the charge developed on the carbonyl oxygen atom. Once inserted in an appropriate thermostable hydrolase scaffold, the resulting enzyme exhibited higher activity for MHET hydrolysis than FAST-PETase. An interesting observation is that a dual enzyme system comprising KL-MHETase and FAST-PETase exhibited a 2.6-fold faster PET depolymerization rate than FAST-PETase alone, demonstrating the possibilities of multienzymatic based treatments of plastic wastes.^60^

### 4.4 Using the Stark Effect to Design Better Dehydrogenases

Boxer and co-workers used the Stark effect to study the electric field on the carbonyl group of the substrate in horse liver alcohol dehydrogenase.^61^ This enzyme catalyzes the reduction of aldehydes/ketones to alcohols by NADH (Figure 8b), which involves the polarization of the carbonyl bond and the increase in the associated bond dipole. The authors found a linear correlation between the electric field, deduced from the shift of the vibrational frequency associated with the carbonyl group stretching, and the activation free energy in wild-type enzyme and several variants that include mutants and also the result of substituting the metal ion present in the active site (from Zn^2+^ to Co^2+^ or Cd^2+^). The experimental work showed that the hydride transfer reaction catalyzed by horse LADH can be improved by mutating the catalytic serine to threonine or by replacing the Zn^2+^ metal ion with Co^2+^. The Stark effect was shown to be an excellent predictor of the catalytic efficiency in LADH variants, demonstrating the importance of electric field effects in the design of improved catalysts. In addition, the authors found that the effects of the single point mutation and ion replacement were additive, which could facilitate the design of more efficient enzymes guided by the electric field effects derived from group contributions.

## 5 Perspectives

The enzyme catalyzed processes discussed in this paper illustrate that electrostatic preorganization and reorganization make major contributions to catalysis in many cases. Based on the results of a series of experimental and QM/MM computational studies, the analysis of the electrostatic potential and the electric field generated by the protein allowed us to illustrate how these concepts work in different enzymes and how they can be used to guide protein design in cases where the electronic density at the transition state differs significantly from that of the reactants. However, while these electrostatic concepts have already been used to improve the design of some biocatalysts, their practical applications are still not widely extended. Some of the difficulties associated with the use of electrostatics to guide the selection of mutations are the long-range nature of these interactions and its dependence on protein dynamics. The mutated amino acid, even if it is located far from the active site, may have a noticeable effect on the electrostatic properties of the active site if its charge distribution differs significantly from that of the original residue. In addition, there is also an indirect effect due to the change in the structure and dynamics of the protein induced by the mutation. This indirect electrostatic effect can be more difficult to reproduce by current simulation techniques because it requires the correct sampling of the conformational spaces available to the enzyme variants. In spite of these difficulties, the usefulness of electrostatic concepts must serve as an incentive to the development of new methodological approaches for their incorporation in the protein design process.

The application of machine learning techniques is sparking a revolution in the field of protein structure prediction and enzyme design. New codes such as AlphaFold2^7^ and RoseTTAFold2^62^ have significantly improved the accuracy of predicted structures from a given sequence. RFdiffusion^6^ employs denoising networks and training on diffusion models to generate plausible structures to accommodate specific active sites. By applying advanced deep learning techniques, these methods predict protein structures with unprecedented accuracy. The challenge for the near future is to combine these methods with the knowledge acquired about enzymatic catalysis, in particular of the electrostatic principles reviewed in this work, to design proteins with the desired catalytic properties. A deep knowledge of the molecular mechanism of the reactions catalyzed by enzymes, including the evolution of the charge density required for the chemical reaction to proceed, which can be acquired by QM/MM methods, appears to be essential to complement automated techniques.

As said, a key point still missing in the machine learning approach to enzyme design is the incorporation of electrostatic properties, from both static (preorganization) and dynamic (reorganization) points of view. The basic approach to protein design is still based on the design of an active site, the anchoring into a protein scaffold that can be selected or generated using deep-learning techniques, and the improvement of the resulting design by means of directed evolution.^41^ However, electrostatic effects are long-range effects, and the full electrostatic potential or electric field on particular positions of the reactive system selected using a mechanistic knowledge is not only due to active site residues but also to residues placed away from the active site. In this sense, the contribution of the protein scaffold to the electrostatic preorganization of the active site and the importance of selecting those scaffolds that contribute favorably to the electrostatic properties in the active site have been emphasized.^46^ Future design strategies should incorporate electrostatic requisites in the selection of the scaffold where the active site should be anchored considering, for example, the contribution to the electric field of protein secondary motifs (such as the dipole moment associated with α-helixes). It has been recently proposed that the electron density of the active site could serve as an indicator for a correct preorganization of the whole enzyme.^63^ A method based in deep neural networks, ProteinMPNN,^5^ could be useful to design protein sequences folding in structures displaying the desired electrostatic properties. ProteinMPNN offers a promising route for optimizing protein properties, showcasing its potential impact on protein engineering and molecular design.

The second principle to be incorporated in future protein designs is the reorganization of the enzyme, a concept related to protein flexibility. Reorganization is needed to maximize the binding of the substrate, but its impact on the activation barrier must be minimized. Mutations not only must be selected to optimize the electrostatic properties to stabilize the reactions TS but also must be filtered out to avoid the population of suboptimal configurations at the reactants state, configurations that require an additional energy cost to progress toward the TS. Molecular simulations have shown that successful directed evolution of Kemp Eliminases not only improves the preorganization of the active site but also creates dynamical networks that facilitate the evolution of the protein toward TS-like configurations.^64^ This means that minimizing the cost associated with the enzyme reorganization should improve the enzymatic designs. Encoding protein dynamics in enzyme design is undoubtedly one of the biggest challenges for the future of the field.^65^

It is finally worth mentioning that a more complete understanding about the role of electrostatic properties in catalysis will be gained from longer and more precise simulations of enzymatic reactions. The holy grail of molecular simulations, the development of accurate yet computational efficient energy functions, could be at hand thanks to the development of machine learning potentials (MLPs)^66−68^ and the combination of them with a molecular mechanics description for larger parts of the systems under study (ML/MM approaches).^69,70^ A correct description of the electronic density of the reactive subsystems and its electrostatic interactions^70^ is a requisite to extract useful information on MLP and ML/MM simulations to guide future enzyme designs.
